# Implementation of Direct-to-Patient Mobile Teledermatology in VA

**DOI:** 10.1007/s11606-023-08480-1

**Published:** 2024-01-22

**Authors:** Sara B. Peracca, Olevie Lachica, Rebecca P. Lamkin, George L. Jackson, David C. Mohr, Heather A. King, John D. Whited, Allene S. Fonseca, Isis J. Morris, Allen L. Gifford, Martin A. Weinstock, Dennis H. Oh

**Affiliations:** 1 Dermatology Service (190), San Francisco VA Health Care System, 4150 Clement Street, San Francisco, CA 94121 USA; 2 Center for Healthcare Organization and Implementation Research, VA Boston Healthcare System, 150 South Huntington Ave, Boston, MA 02130 USA; 3 Center of Innovation to Accelerate Discovery and Practice Transformation (ADAPT), Durham Veterans Affairs Health Care System, 508 Fulton Street, Durham, NC 27705 USA; 4 Peter O’Donnell Jr. School of Public Health, University of Texas Southwestern Medical Center, 5323 Harry Hines Boulevard, Dallas, TX 75390 USA; 5 Department of Health Law, Policy & Management, School of Public Health, Boston University, 715 Albany Street, Boston, MA 02118 USA; 6 Department of Population Health Sciences, Duke University, 215 Morris Street, Durham, NC 27701 USA; 7 Division of General Internal Medicine, Duke University School of Medicine, 6301 Herndon Road, Durham, NC 27713 USA; 8 Department of Dermatology, Wayne State University, 18101 Oakwood Boulevard #402, Dearborn, MI 48124 USA; 9 Section of General Internal Medicine, Boston University Chobanian & Avedisian School of Medicine, 72 E Concord Street, Boston, MA 02118 USA; 10 Department of Dermatology and Epidemiology, Brown University, 593 Eddy Street, Providence, RI 02903 USA; 11 Center for Dermatoepidemiology, Providence VA Medical Center, 830 Chalkstone Avenue, Providence, RI 02908 USA; 12 Department of Dermatology, University of California San Francisco, 1701 Divisadero Street, San Francisco, CA 94115 USA

**Keywords:** mobile teledermatology, direct-to-consumer teledermatology, asynchronous care, implementation science, dermatology, telemedicine

## Abstract

**Background:**

Innovative technology can enhance patient access to healthcare but must be successfully implemented to be effective.

**Objective:**

We evaluated Department of Veterans Affairs’ (VA’s) implementation of *My VA Images*, a direct-to-patient asynchronous teledermatology mobile application enabling established dermatology patients to receive follow-up care remotely instead of in-person.

**Design /Participants/Approach:**

Following pilot testing at 3 facilities, the app was introduced to 28 facilities (4 groups of 7) every 3 months using a stepped-wedge cluster-randomized design. Using the Organizational Theory of Implementation Effectiveness, we examined the app’s implementation using qualitative and quantitative data consisting of encounter data from VA’s corporate data warehouse; app usage from VA’s Mobile Health database; bi-monthly reports from facility representatives; phone interviews with clinicians; and documented communications between the operational partner and facility staff.

**Key Results:**

Implementation policies and practices included VA’s vision to expand home telehealth and marketing/communication strategies. The COVID-19 pandemic dominated the implementation climate by stressing staffing, introducing competing demands, and influencing stakeholder attitudes to the app, including its fit to their values. These factors were associated with mixed implementation effectiveness, defined as high quality consistent use. Nineteen of 31 exposed facilities prepared to use the app; 10 facilities used it for actual patient care, 7 as originally intended. Residents, nurse practitioners, and physician assistants were more likely than attendings to use the app. Facilities exposed to the app pre-pandemic were more likely to use and sustain the new process.

**Conclusions:**

Considerable heterogeneity existed in implementing mobile teledermatology, despite VA’s common mission, integrated healthcare system, and stakeholders’ broad interest. Identifying opportunities to target favorable facilities and user groups (such as teaching facilities and physician extenders, respectively) while addressing internal implementation barriers including incomplete integration with the electronic health record as well as inadequate staffing may help optimize the initial impact of direct-to-patient telehealth. The COVID pandemic was a notable extrinsic barrier.

**Clinical Trials Registration:**

NCT03241589

## INTRODUCTION

Telehealth, including teledermatology, is an important tool for improving access to healthcare.^1, 2^ Asynchronous or store-and-forward teledermatology utilizes relatively low-cost, simple equipment that can be easy to use while providing clinically useful images. Additionally, the asynchronous nature allows it to be more flexibly integrated into busy clinical practices of both referring clinicians and dermatologists. The Department of Veterans Affairs (VA) operates one of the largest and longest running consultative asynchronous teledermatology programs, primarily reliant on in-clinic imaging of patients.^3–5^ The process, facilitated by its enterprise-wide electronic health record (EHR), is typically initiated by primary care providers who, upon identifying a patient with a skin concern, order a teledermatology imaging consult. Trained imagers respond by generally requiring the patient to come to the clinic for imaging. Images of the skin problem and its associated history are then uploaded into the EHR and a teledermatology reading consult is placed, alerting a VA dermatologist to review and document evaluation of the case. The alerted referring provider then conveys the consult results to the patient and executes any recommendations.

**Figure 1:**
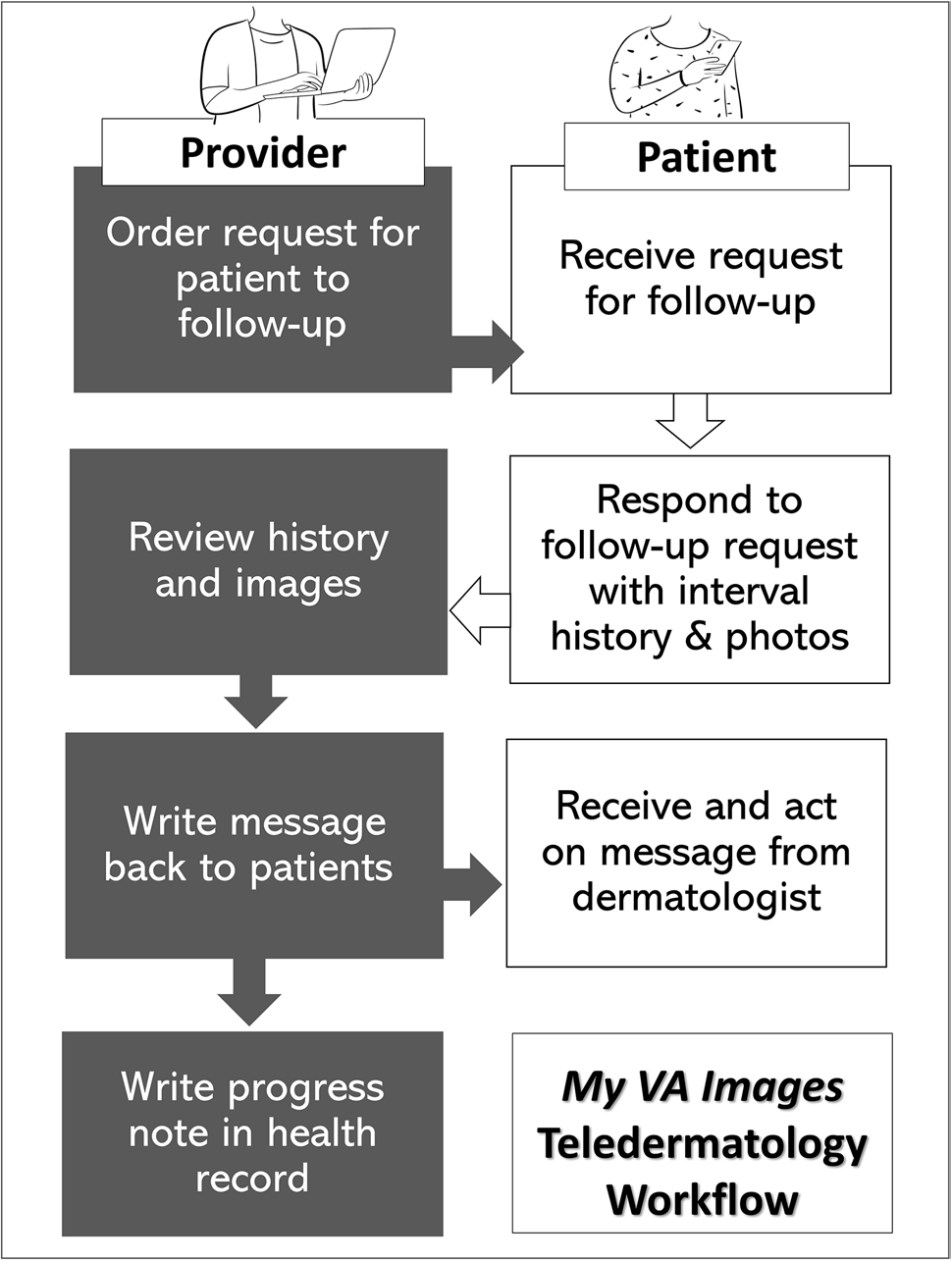
Mobile teledermatology workflow. VA clinicians use *VCM* to create teledermatology requests in *MVAI* for patients, to evaluate patient submissions, to give patients feedback and instructions, and to document the work in the EHR. Patients use *MVAI* to respond to requests for photos and history, and to read the dermatologists’ opinion and recommendations.

Direct-to-patient forms of asynchronous teledermatology have also emerged, allowing patients to submit their own skin photos and to communicate with dermatologists directly.^6, 7^ VA’s Office of Connected Care (OCC) identified the need and developed its own patient-facing asynchronous mobile telehealth application, *My VA Images* (*MVAI*), to mediate direct-to-patient telehealth, including a teledermatology-specific functionality allowing VA dermatologists to request eligible established patients to follow-up remotely. Upon initiation of a request by a dermatologist using the clinician-facing telehealth portal, *Virtual Care Manager* (*VCM*), which is distinct from VA’s EHR, *MVAI* prompts patients to submit interval skin history and guides them in taking images of specific anatomic sites (Figure 1). Dermatologists then use *VCM* to review patient-submitted data, document the clinical progress note and encounter in the EHR, and asynchronously communicate findings and instructions directly to patients through *MVAI*.

**Figure 2:**
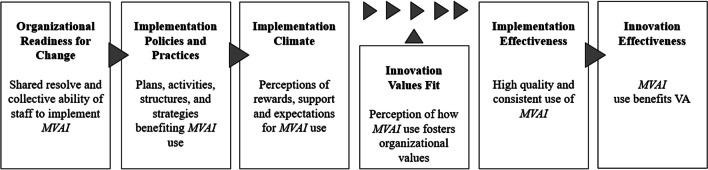
Organizational Theory of Implementation Effectiveness. Innovation effectiveness is dependent on OTIE constructs.

Despite teledermatology’s growth, particularly during the COVID-19 pandemic, there has been little systematic effort to study and guide mobile teledermatology implementation according to established theories. Expanding on initial work by Klein and Sorra, the Organizational Theory of Implementation Effectiveness (OTIE) has been helpful in understanding integration of new technologies in healthcare.^8–10^ OTIE posits that an innovation’s impact arises from implementation effectiveness, which in turn is a result of an organization’s readiness for change (ORC), which drives its policies and practices, the climate arising from those policies and practices, and the extent to which users of the intervention perceive that it fulfills their values (Figure 2).

As part of a larger study examining the effect of teledermatology mobile apps on access, we examined *MVAI*’s introduction to multiple VA facilities.^11^ We previously reported that ORC scores appeared to correlate with successful *MVAI* activity, and we identified antecedent factors associated with *MVAI* readiness.^12^ The current work examines the other downstream OTIE components. The results systematically document implementation of a new technology in the VA’s asynchronous teledermatology ecosystem and identify both challenges and opportunities, even for an organization with relatively mature telehealth operations.

## METHODS

The study was approved by San Francisco, Durham, Providence, and Boston VA Medical Center Institutional Review Boards.

### Study Design

The study of implementation was part of a stepped-wedge cluster-randomized trial to understand the impact of VA mobile teledermatology applications, previously described in detail.^11^ Inclusion criteria required facilities to have prior asynchronous teledermatology experience, defined as being ≥9% of all fiscal year (FY)18 dermatology encounters. The exclusion criterion was lack of an in-person dermatology clinic in FY18. Following technical pilot testing at 3 facilities, 4 groups of 7 facilities (28 total) were sequentially exposed to *MVAI* every 3 months. Groups were balanced to have facilities with comparable geographic and medical complexity distribution.^11^ The 3 pilot facilities, which also fit the inclusion and exclusion criteria, continued to use *MVAI*, contributing data for the duration of the study.

### Data Collection

We used qualitative and quantitative data throughout the study to understand the implementation. To allow facilities time to learn about *MVAI* and organize local implementation, data collection commenced 3 months following each facility’s initial exposure to *MVAI* and continued through the end of September 2020. Data included the following: (1) Quantitative data from VA’s corporate data warehouse (CDW) which hosts data from VA’s EHR, as well as from VA’s mobile health database which tracks mobile device activity. We examined total number of attempted and completed encounters and of each type of clinician submitting these encounters from May 2019 to September 2020 for the 3 pilot and 28 randomized facilities; (2) bi-monthly written reports from 19 facilities completed by consenting telehealth and clinical leads between September 2019 and November 2020 using links in email invitations. Reports included responses to open- and closed-ended questions assessing implementation and sustainment, including facilitators and barriers, concerns of key stakeholders about resources and support, reports to leadership, and self-reported ratings of implementation progress on a 10-stage scale associated with pre-implementation (discussion and initial engagement), implementation (resource availability), and sustainability (competency and dissemination) based on concepts in the Stages of Implementation Completion framework (see Supplementary Materials);^13–15^ (3) 12 interviews with clinical staff, clinical leads, and operational facilitators, guided by OTIE concepts, at the pilot facilities midway through the study, and with 7 dermatologists and 3 support staff at 4 additional facilities and one pilot facility at study’s end; and (4) field notes collected throughout the study, documenting conversations with and emails from facility staff at all exposed facilities and question/answer sessions during OCC webinars.

### Quantitative Data Analysis

We matched encounter data from CDW to *MVAI* activity in VA’s Mobile Health database to identify and verify completed requests and instances where app use deviated from intended implementation. Univariate and bivariate statistics of VA CDW encounter data and quantitative data in the bi-monthly reports were performed regularly throughout the study.

### Qualitative Data Analysis

Interviews were recorded, transcribed verbatim, and summarized using rapid analysis techniques.^16^ Open and focused coding combined with matrix techniques facilitated analysis and presentation enabling us to examine commonalities and differences.^17–19^ Core concepts associated with OTIE were identified, including whether these positively or negatively impacted implementation effectiveness. The matrices, with data from the multiple qualitative sources, enabled (1) grouping by characteristics (facility, region, staff type), (2) OTIE constructs (implementation policies and practices, climate, values fit, implementation effectiveness), and (3) time (pre/post COVID-19, changes over time).

## RESULTS

### Implementation Policies and Practices

Consistent with VA’s strategic goal to provide telehealth in the home, OCC began *MVAI* development in 2015, engaging multiple specialty clinicians, including dermatology, and individuals from information technology (IT), clinical informatics, business, and telehealth leadership. Upon successful pilot testing for technical functionality between May and June 2019 at 3 pilot sites, *MVAI* was additionally released to groups of 7 new facilities every 3 months beginning July 1, 2019, for a total of 4 groups and 28 facilities*.*^11^ Release constituted the opportunity to use *MVAI*, but OCC did not require facilities to do so and no incentives were offered other than the apps’ intrinsic merits.

To alert and engage key stakeholders, OCC established multiple mechanisms to introduce and monitor MVAI. Two weeks before official release at each facility, OCC sent a brief email to inform the dermatology chief. On the day of release, OCC sent official emails to facility leaders: the dermatology chief, telehealth coordinator, chief of staff, and director, as well as the regional network telehealth coordinator and any other known facility teledermatology champions dedicated to advocating for *MVAI*. The email provided (1) introductory information; (2) a checklist of tasks aligned with VA’s informatics policy and practice that were necessary to enable interface with the VA EHR; (3) electronic copies of promotional/instructional materials for clinician and patient end-users; (4) links to online videos demonstrating the workflow; and (5) invitations to live online OCC training webinars and biweekly check-in sessions. At least one key individual from each of the exposed facilities attended a webinar, confirming receipt of the emails by each facility. Field notes from webinars and responses to the emails captured a general sense of excitement among participants to implement *MVAI*, though concerns and skepticism were occasionally expressed*.*

Clinical champions, who by definition were interested in and dedicated to advocating for change,^20^ facilitated implementation of policies and practice locally. Champions were largely dermatologists or telehealth leads who self-selected to help complete the set-up process, encourage others to try *MVAI*, and create systems to provide marketing/training materials to patients. Of the 19 facilities completing at least one bi-monthly report (a 61% response rate), 6 documented that support from clinical champions was instrumental for implementation. Field notes also captured the importance of clinical champions; at one facility, lack of senior leadership support almost led to program discontinuance but for the champion stepping in.

Anticipating the need to capture clinical workload and to facilitate tracking *MVAI* usage according to VA’s usual practice, OCC created traditional clinical informatics tools and new mechanisms in *VCM* and other databases to monitor key milestones for *MVAI* encounters, which were distinct from informatics processes and required separate user workflows.

Reception of these policies and practices were mixed. Once exposed to *MVAI*, interviewed clinicians found it user-friendly and the workflow straightforward, “It was easy to use…easy to initiate,” “nice to email securely with patients….” Clinicians appreciated *MVAI*’s intended use cases for both short-term (e.g., “to see how an incision is healing 1-3 weeks later”) and longer-term follow-ups (e.g., for patients with rashes and on biologics). They also found it beneficial not just as a substitute for an in-person visit but to supplement a separate VA-developed app (*VA Video Connect* or *VVC*), mediating home video visits. In such cases, *MVAI* contributed “clearer images for diagnoses.” When clinicians mentioned image quality, most stated that patient-submitted photos “were very good.” Nevertheless, one facility reported image quality concerns, preferring VA’s standard consultative teledermatology utilizing in-clinic imaging.

*MVAI*’s incomplete integration with VA’s pre-existing EHR, such as the inability to upload images directly to the EHR, concerned some clinicians. For example, *VCM* enabled clinicians and staff to track *MVAI* cases but required an extra step outside of the usual EHR workflow. Clinicians’ frustration that patients often did not respond to *MVAI* requests was an additional barrier identified in all qualitative sources.

All facilities experienced some technical difficulties with *MVAI*. Uncoordinated system upgrades to VA’s EHR and mobile health infrastructure, which were each overseen by separate VA offices, created brief user problems. Idiosyncrasies with local IT infrastructure ultimately led two facilities to discontinue *MVAI* use.

### Implementation Climate

*MVAI* was introduced at facilities that were specifically chosen to have expertise in teledermatology and telehealth in general (see the “Methods” section). Thus, the majority of administrative and clinical support staff, involved in preparing the EHR for *MVAI*, reported in bi-monthly reports no concerns with their involvement or resources available. Interestingly, the fraction of staff with concerns was greater at facilities introduced to *MVAI* after the onset of the COVID-19 pandemic (40%) than those exposed pre-pandemic (≤33%). Some concerns arose from misunderstandings, such as when one facility delayed *MVAI* implementation because it believed *MVAI* was not an approved protocol during the pandemic.

Patient eligibility was a significant factor in determining climate. Since additional clinic time was not allocated to screen for patient eligibility, use *VCM*, or educate patients, interviewed clinicians viewed *MVAI* as a disruption. It was “one more thing… More work without taking away anything.” Highlighting that only ~50% of patients were already enrolled in VA’s mobile health program and that not every patient owned or was proficient with a mobile device, one interviewed clinician stated that it was difficult to “find the ideal patient.” To address these concerns, some facilities used administrative data to pre-identify eligible patients or utilized support staff to assist dermatology clinicians with *VCM by* creating *MVAI* requests and to assist patients with *MVAI*.

**Figure 3:**
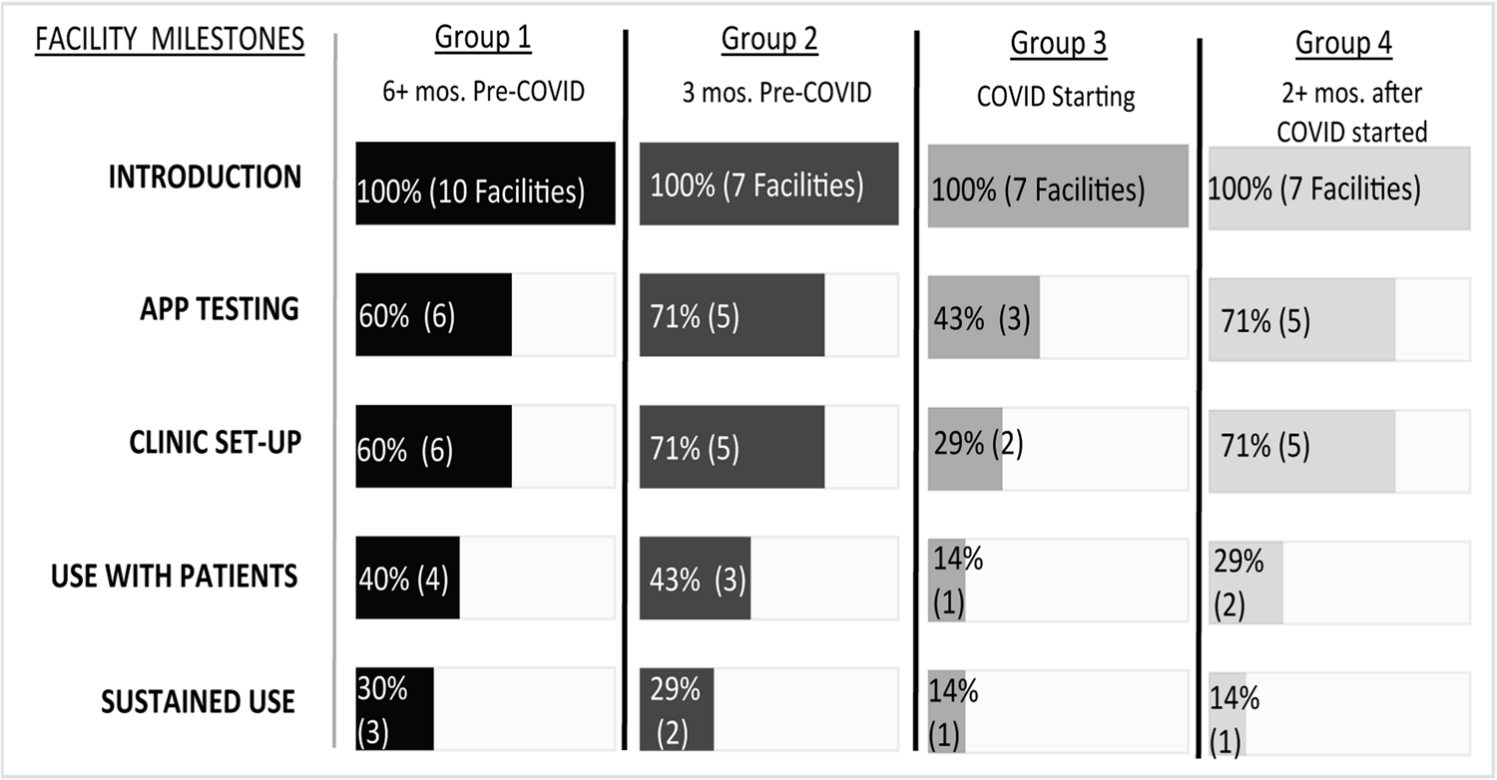
Implementation tracking for *MVAI*. Facilities were grouped into those exposed to *MVAI* 6 months or more prior to the onset of the COVID-19 pandemic (group 1); 3 months prior (group 2); at the start of the pandemic (group 3); and 2 months after (group 4). Key milestones for facilities were documented. Sustainment was defined as a facility having a minimum of 2 patients complete a visit and demonstrable evidence of app usage at the end of the study.

The COVID-19 pandemic significantly affected implementation climate. For example, the timing of facilities’ exposure to the app correlated with the rate of achieving implementation milestones (Figure 3). Nearly twice as many facilities introduced to the app pre-COVID achieved each milestone relative to post-COVID facilities, and differences in sustaining use were noticeable. Multiple reasons appeared to explain this phenomenon.

First, apart from the time-consuming demands of identifying patients noted above, inadequate staffing itself was a limitation that the pandemic magnified. Nine facilities reported staff shortages, contributing to delays or stoppage in *MVAI* use. Field notes captured that a facility attempted to implement *MVAI* but loss of one dermatologist precluded its use. Qualitative data from all sources revealed some concern about the lack of on-site staffing to assist patients. In-person clinic closures due to COVID-19 additionally burdened staff who spent extra time sending brochures/instructions to patients instead of giving them in-person. The common use of part-time physicians also made it difficult for facilities to train all clinicians together with the new workflow (“All seven of us doctors are part time…hard to find time to train”).

Second, competing demands existed. Qualitative data sources from all facilities captured telehealth staff narratives about being tasked with setting up other programs in response to COVID-19, increasing demands on their time and delaying preparations. Facilities also reported either increasing their consultative teledermatology program capacity and/or trying other remote options such as *VVC* or encrypted emails to receive photos directly from patients at home, both of which were strongly promoted by VA during these years. One Chief of Medicine said using “the app would take away from … clinical obligations.” All qualitative data sources indicated that clinicians experienced increased demands or reassignments associated with COVID-19. One clinician stated, “I can’t run two new programs at the same time.” Clinicians had less time to train for the new workflow.

Attitudes of stakeholders are an important factor in determining implementation climate. Concerns regarding support and resources for the mobile apps from dermatology chiefs, nurses, and facility leadership decreased after COVID-19’s onset, though differences among groups existed. Of 14 facilities responding to a question on the bi-monthly report about whether dermatology clinicians had concerns, 4 (29%) identified major concerns in at least one, though this response did not correlate with when a facility was exposed to *MVAI*. In contrast, at 4 of 12 facilities responding to a question on the bi-monthly about whether senior leadership had concerns, 3 were among the groups exposed pre-pandemic. Senior facility leadership’s engagement, often important in funding continued operations, is instrumental in creating a strong implementation climate.^21^ However, using reporting as an indicator of engagement, we found no association between reporting to leadership and use of *MVAI* at their facility.

### Fit to Values

Possibly reflecting VA’s strong mission to serve Veterans, bi-monthly reports and interviews elicited that dermatology clinicians were primarily interested in adopting *MVAI* to improve access, to increase patient convenience, and to make in-person appointments available. They felt *MVAI* would particularly benefit patients who lived far from a dermatology clinic or who did not want to wait for the next in-person visit.

COVID-19 influenced facilities’ interest in *MVAI*. Many dermatology clinicians were “committed to implementing whatever telehealth resources were available.” *MVAI* enabled clinicians to “manage simple patient concerns” remotely to address “the growing backlog of patients” due to appointment cancellations.

### Implementation Effectiveness

**Figure 4:**
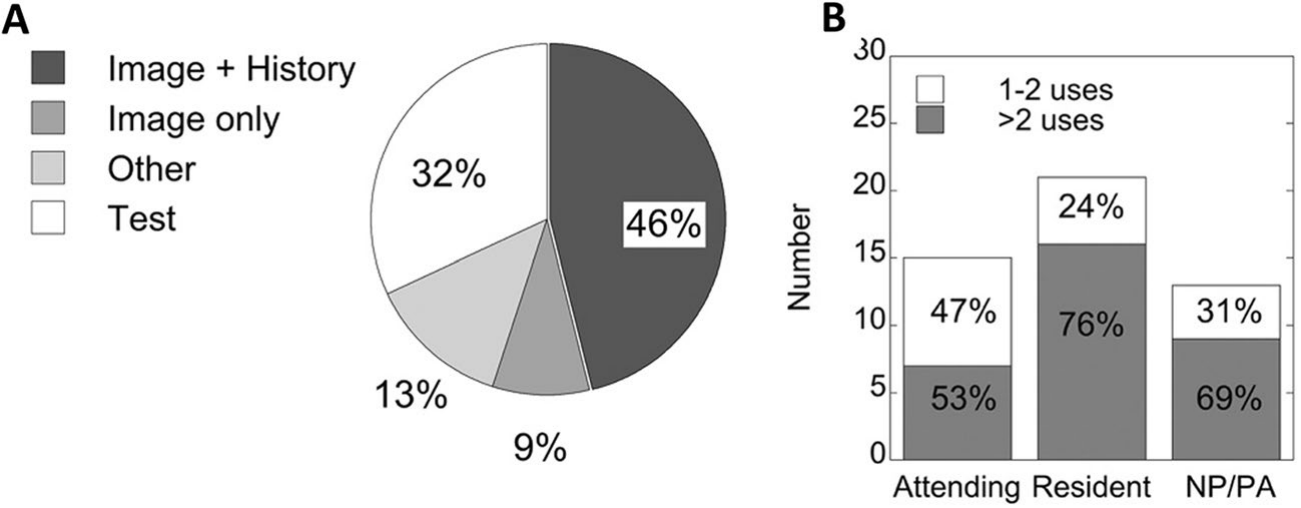
*MVAI* usage. Actual *MVAI* usage reflected by CDW and VA Mobile Health encounter data. Manual inspection was used to categorize encounters by **A**) test patients (unshaded), and actual patients (shaded areas), and **B**) low- and high-end clinician user types.

During the 17-month study period, 10 (32%) facilities used *MVAI* to care for 466 unique patients with a total 474 encounters. Nineteen of 31 facilities (61%) implemented *MVAI* by setting up clinics according to CDW data: 10 of the 19 facilities used *MVAI* for actual patient care by completing encounters in the EHR; 9 tested it only, reflected by VA mobile health data activity but no formal EHR encounters. Two hundred twenty of 474 (46%) encounters occurred at 7 facilities using *MVAI* as originally intended with patient-submitted images and history. Six facilities generated *MVAI* teledermatology encounters technically correctly but deviated by requesting patient-submitted photos without history (9% of all visits)—one facility reported that they adapted *MVAI* to augment video visits with *VVC*, suggesting others did as well. Testing accounted for 32% of usage, reflecting intent to implement, while the remaining *MVAI* encounters (13%) reflected various errors in app usage (Figure 4A).

Based on CDW data, use varied by clinician type. An adaptive strategy used by some facilities was to encourage use by residents, nurse practitioners, and physician assistants (NP/PAs), with NP/PAs using MVAI more frequently than attendings. Most *MVAI* clinicians were resident/fellow trainees, followed by dermatology attendings (Figure 4B). Consistent with these results, two VA facilities that were academic teaching affiliates contributed 84.4% of all *MVAI* encounters. However, the additional time required to train residents about *MVAI* was also identified as a burden by 4 facilities in bi-monthly reports. 

**Figure 5:**
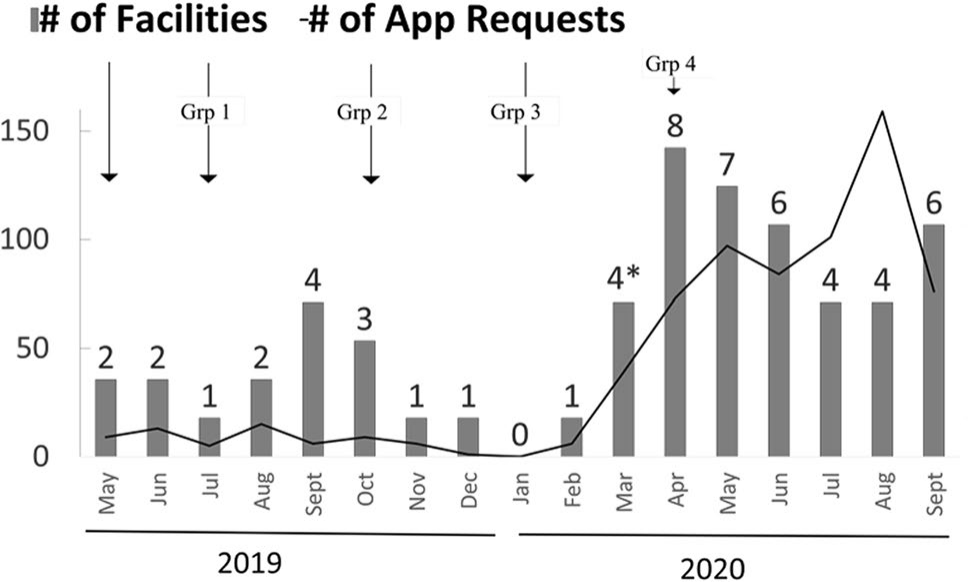
Time-dependence of *MVAI* usage. *MVAI* piloting was followed by rollout according to a cluster-randomized stepped-wedge design over 17 months. Bars represent the number of facilities recording any *MVAI* requests for real patients with actual number of facilities stated above each bar. The solid line represents actual number of *MVAI* requests ordered over all exposed sites. Arrows denote when groups of facilities were exposed to MVAI, starting with pilot sites in May 2019. Asterisk denotes the shelter-in-place orders at the start of COVID-19 pandemic.

**Figure 6:**
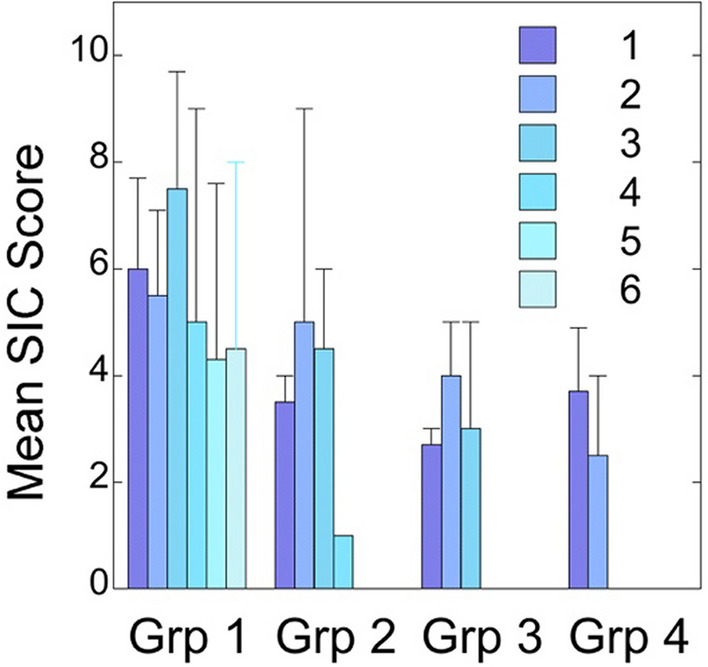
Stages of implementation completion (SIC). Bi-monthly reports were used to assess SIC scores for each group (Grp) or cohort of facilities throughout the study period. Mean scores and standard deviations are plotted for each group for each reporting period following exposure to *MVAI*. Note that because each group was surveyed only after *MVAI* exposure, Group 1 facilities had the opportunity to complete up to 6 bi-monthly reports, whereas subsequent groups had progressively fewer opportunities. A higher score denotes greater implementation completion.

Although not universal, clinicians increasingly used *MVAI* as in-person clinics closed due to COVID-19 (Figure 5). To understand the variability in use, we examined self-reported implementation progress, and found that facilities with lower initial implementation stages were associated with the onset of COVID-19 (Figure 6). Of 8 facilities that provided at least one bi-monthly report after the pandemic started, 4 reduced their implementation completion stage, 2 stayed the same, and 2 increased their stage.

Of the 10 facilities that used *MVAI* with patients, 7 sustained usage through the end of the study period, with 3 stopping due to technological barriers or leadership prioritizing other goals.

## DISCUSSION

Home-based telehealth has had an increasing presence in VA and in healthcare generally.^22–27^ Direct-to-patient asynchronous teledermatology has been studied in research settings with defined diseases and populations.^28–30^ However, differences in how innovative interventions are implemented among different organizations may exist.^31, 32^ Here we attempt to systematically study implementation of direct-to-patient asynchronous teledermatology in a natural setting within a large national healthcare system using the context of implementation theory. Components of the OTIE have been used to understand a variety of health interventions, though its application to telehealth has been limited.^12, 33–35^ This work examines OTIE elements downstream of ORC to better understand *MVAI*’s varied implementation effectiveness.

As a federal integrated healthcare system whose facilities, workforce, and patients share many commonalities, VA has potential strengths in implementing new healthcare technology.^36^ Telehealth technology such as *MVAI* is governed by overall VA IT and telehealth policies and guidelines. However, individual facilities can still differ in local policies and priorities, leading to heterogeneous implementation microclimates.^37, 38^ We have previously reported ORC varied among our pilot sites, and this result presaged the implementation effectiveness heterogeneity we observed in this study.^12, 35^

*MVAI* was wholly developed by and for the healthcare system that implemented it, and thus aligned with the perspective of most employees that *MVAI* fit with VA values. Knowledge of VA’s mission, policies, structure, and clinical practice idiosyncrasies was largely incorporated into *MVAI*’s design, including partial integration with the VA’s EHR. Therefore, previously identified institutional barriers associated with mobile teledermatology such as lack of compatibility with the EHR and the lack of legal and regulatory policies to protect patient privacy and define practitioners’ risk were not of concern*.*^35, 39, 40^ The significance of incomplete integration with the EHR, however, may have been underestimated as an important factor in implementation practice. Potential users faced the burden of learning about and using an additional parallel platform. VA has subsequently improved *MVAI*’s integration for end-users by creating quality improvement tools that are more familiar and accessible to staff responsible for monitoring, and by enabling images to be uploaded into the EHR to ease the integration barrier.

Implementation plans recognized the heterogeneity among facilities by providing guidance rather than proscriptive mandates for many implementation decisions and by encouraging local decisions such as which dermatology clinics would deploy *MVAI* first. As a result, some facilities with supportive implementation climates innovated adaptations that fit their needs and available resources, such as by using *MVAI*, not as a standalone teledermatology platform as it was originally intended, but as a complement to *VVC.* Facilities also innovated by leveraging non-dermatologists to screen for eligible patients. These adaptations may be useful to incorporate in future *MVAI* marketing.

Implementation did not of course anticipate the onset of the COVID-19 pandemic, which had significant effects on implementation climate. Ironically, while telehealth was a critical healthcare delivery strategy during the pandemic, which increased dermatologists’ interest in *MVAI*’s potential value, numerous competing demands on staff by VA, including promotion of other telehealth pathways, also increased during the pandemic, leading to a fragmented or distracted implementation climate at some facilities.^22, 41^ Thus, we observed that facilities that had been exposed to *MVAI* before the pandemic had implementation climates that were better prepared for *MVAI* usage and more likely to achieve key milestones.

Staffing practices emerged as an important consideration, consistent with prior studies.^42^ Facilities that were academic affiliates who had resident trainees accounted for most *MVAI* activity. NP/PAs were also important end-user adopters of *MVAI*. These types of clinicians perhaps had more capacity than part-time attending dermatologists to accommodate the initial extra workload of learning about and acclimating to new processes. It may be useful to target these types of clinicians as an implementation practice in future telehealth programs to optimize initial adoption. The importance of non-clinician support staff to assist with performing administrative and routine tasks including determining patient eligibility, entering *MVAI* requests into *VCM*, and distributing education and instructions to patients was also an important practice contributing to a positive implementation climate.

Determining eligibility proved to be a significant systemic policy and practice barrier, as security policies required Veterans to have been enrolled in VA’s mobile health program. Only about half of all Veterans were eligible during the study period. On the other hand, concerns reported elsewhere regarding addressing multiple skin disorders, gathering adequate medical history, the possibility of misdiagnosis, and the limited ability to counsel patients with sensitive or complicated diagnoses were not raised by our study participants, perhaps because *MVAI* was marketed as a tool for follow-up of established rather than new patients.^43–46^

Leadership during implementation emerged as another climate facilitator, as has been well-documented.^12, 36, 47, 48^ We found clinical champions are important in the initial phases of implementation, but for sustained use senior leaders’ support results in a stronger implementation climate. With good innovation-values fit, facilities with leaders that created strong implementation climates experienced consistent use. Our measure of senior leadership involvement was not associated with sustainment, possibly because of the relatively small sample (5) or the brief duration of our study. Additionally, while *MVAI* was designed to be a general direct-to-patient telehealth tool, its implementation at our study sites was restricted to dermatology. Thus, dermatology clinical champion efforts were largely isolated and could not synergize with activities of champions in other specialties as part of a facility-wide campaign. Similarly, while VA’s OCC leadership supported *MVAI*’s limited release, *MVAI* did not benefit from national marketing, training resources, and interactions among the community of practitioners that normally accompany a VA-wide rollout of a new telehealth program.^23, 49, 50^

## LIMITATIONS

Facilities were selected based on the requirement for significant pre-existing experience with consultative teledermatology and may not be representative of all VA facilities. Variable response rates with questions on the bi-monthly reports may have influenced the reproducibility of results. COVID-19 reduced the uniformity of the implementation environment due to our time-dependent stepped-wedge design. Finally, this study did not directly examine implementation from the important perspective of patients.

## CONCLUSION

While there was success in implementing *MVAI* at some sites, the heterogeneity of implementation illustrates challenges in deploying new technologies supplementing usual healthcare workflows. These challenges occurred despite VA’s highly functioning integrated national healthcare system and its culture of supporting telehealth innovation. As usage among staff varied, targeting higher-end users such as trainees and NP/PAs may be a useful initial strategy for future telehealth implementation. Leveraging other support staff to assist with usage can also be an important facilitator to mitigate competing demands. While some barriers arose due to intrinsic factors associated with using new technology and workflows, others occurred due to extrinsic factors, specifically the effect of the COVID-19 pandemic. With increasing use of technology in healthcare, addressing intrinsic barriers through technology and implementation design are important to minimize the impact of extrinsic factors.

## Declarations

### Disclaimer

The views expressed in this report are those of the authors and do not reflect the position or policy of the Department of Veterans Affairs or the United States government.
